# Value of Adaptive Trials and Surrogate Endpoints for Clinical Decision-Making in Rare Cancers

**DOI:** 10.3389/fonc.2021.636561

**Published:** 2021-03-08

**Authors:** Andriy Krendyukov, Sanjay Singhvi, Markus Zabransky

**Affiliations:** ^1^ Apogenix AG, Heidelberg, Germany; ^2^ System Analytic, London, United Kingdom; ^3^ Sandoz GmbH, Holzkirchen, Germany

**Keywords:** adaptive clinical trial, innovative medicinal product, rare cancer, surrogate clinical endpoint, clinical decision

## Abstract

Despite high-level endorsement, the number of adaptive Phase II/III trials in rare cancers needs to be improved, with better understanding of their value for clinical decisions in daily practice. This paper describes approaches to trial design in rare cancers, which has been supplemented by a search of ClinicalTrials.gov for adaptive trial designs in rare cancer. In addition, an online survey of 3,200 oncologists was conducted. Practicing physicians were questioned on the importance of different evidence levels, types of adaptive trial design, and categories of surrogate endpoints for clinical decision making. The results of the online survey revealed that evidence from Phase II/III trials with an adaptive design and relatively small sample size was considered high value in rare cancer by 97% of responders, similar to the randomized controlled trial rating (82%). Surrogate clinical endpoints were considered valuable alternatives to overall survival by 80% of oncologists. Preferred adaptive designs were futility analysis, interim analysis, adaptive sample size, and adaptive randomization. In conclusion, rare cancer oncologists rate evidence from adaptive clinical trials with as high a value and importance for clinical decision making processes as conventional randomized controlled trials. All stakeholders have a vested interest in advances in clinical trial designs to ensure efficient and timely development of innovative medicinal products to allow more patients faster access to the pivotal treatment.

## Introduction

Rare cancers have been variously defined as those with a prevalence of fewer than five cases out of a population of 10,000 (European definition) and cancers with fewer than 15 cases per 100,000 people per year (US National Cancer Institute definition) (1). In an effort to standardize the reporting and treatment of rare cancers and allow global comparisons a consortium from the European Union, Surveillance of Rare Cancer in Europe (RARECARE) put forward a revised definition of rare cancers as those with fewer than six cases per 100,000 people per year (2). A list provided by the RARECAREnet project, which is derived from the data of 94 population-based cancer registries from 24 European countries, indicates there are many hundreds of rare cancers that combined make a large contribution to overall cancer incidence (3). Rare cancers accounted for 24% of all cancers diagnosed in Europe during 2000-2007 affecting around 4.3 million people (4), albeit with disparities in both incidence and survival between different countries (5), and 20% of cancers diagnosed in the USA during 2009-2013 (6). Furthermore, while individually, each of the 198 currently identified rare cancers is considered ‘rare’, collectively they account for around 22% of all cancer cases diagnosed in Europe each year, higher than any single common cancer.

Specific challenges posed by rare cancers include late or incorrect diagnosis and lack of access to appropriate therapies and clinical expertise. The development of innovative medicinal products (InMPs) for rare cancers faces many challenges including difficulties in recruiting adequate numbers of patients from a very small and heterogeneous patient population, limited knowledge of disease natural history, and from a pharmaceutical company point of view: high financial investments, extensive development times, and significant risk of potential failure (7).

Many rare cancers affect less than one in 100,000 people, are life-threatening and progress rapidly making them difficult to study in conventional randomized controlled trials. This is intensified in pediatric populations in whom cancer is rare in general and rare pediatric cancers are even rarer (8). Alternative approaches to clinical trial design are therefore required that allow cost-effective, well-controlled, and relevant analyses to assess treatment effects in small, heterogeneous populations on a shorter time scale (9). Adaptive trials, with or without the use of surrogate endpoints, may represent a powerful alternative to conventional randomized controlled trials for InMP development in oncology and, in particular, when targeting rare cancer patients (9). However, despite initiatives by European and US regulatory agencies and recommendations by competent authorities and medical societies, an increase in the number of adaptive Phase II/III trials in rare cancers has not occurred (10). While a few studies have examined challenges in the field of rare cancers and made suggestions for how to conduct trials in rare diseases (11, 12), none have focused on the benefits of adaptive trials for practicing oncologists. This paper aims to examine the potential value of adaptive clinical trials and surrogate clinical endpoints for clinical decisions in rare cancer. The findings are complemented by original data from a survey of oncologists practicing in rare cancer to highlight the perceived value of these trials to the oncology community and encourage their wider implementation.

## Approaches to Trial Design in Rare Cancers

### Strategies to Accelerate InMP Development Timelines

In the heavily regulated pharmaceutical environment, it can take a decade or more between the first synthesis of a new active substance and the InMP reaching the market (13). Many may fail to demonstrate clinically relevant efficacy outcomes or are associated with serious adverse effects before ever reaching this stage. Analysis of new therapeutic agents approved by the US Food and Drug Administration (FDA) between 2009 and 2018 estimated that in oncology, the percentages of FDA approvals were 3.4% for therapeutic agents entering Phase I, 6.7% for those entering Phase II, and 35.5% for those entering Phase III (14). To avoid delays in patients gaining access to InMPs and to optimize the potential revenue for manufacturers (15), it is in the interest of both pharmaceutical companies and regulatory agencies to ensure pivotal trials are completed as rapidly as possible with minimal risk. Significant efforts have been made by the European Medicines Agency (EMA) and US FDA to accelerate the development, review, and approval of InMPs for serious and life-threatening conditions in the form of breakthrough therapy designation by the FDA (16), or priority medicine designation by the EMA (17). Broader implementation of adaptive clinical trials and surrogate outcomes are two further potential tools to speed up the InMP development process.

### Adaptive Clinical Trials

Clinical trials with an adaptive design are defined by the FDA as those that allow for prospectively planned modifications to one or more aspects of the design based on accumulating data from subjects in the trial (18). During a clinical trial, information is amassed that was not available when the trial began and that reduces uncertainty regarding optimal treatment approaches, for example, optimal dose, duration of treatment, and target patient population. Adaptive clinical trials are designed to take advantage of this information by allowing review of data at a prespecified time point during the trial. These data may then be used to make predefined adaptations to key parameters without undermining the integrity and validity of the results; all planned adaptations are defined a priori during trial design and before the trial begins. Potential advantages offered by trials with an adaptive design are illustrated in **Supplementary Table 1** (18, 19).

Adaptive design categories have been defined to help distinguish the methods available, but there is some degree of overlap and trials may also combine more than one adaptive design method (20). Common designs include those that allow for adaptively assigning doses (escalation/de-escalation, to assess dose-outcome relationships); early stopping of the trial for toxicity, efficacy, or futility; dropping or adding new treatment arms; using a seamless phase transition to permit continuation from one phase to another (e.g. Phase II to Phase III); response adaptive randomization techniques (e.g. play the winner, drop the loser) in which the chance of a newly-enrolled patient being assigned to a treatment arm varies over the course of the trial based on accumulating outcome data; sample size re-estimation; and biomarker-guided treatment allocation. Major adaptive design methods commonly employed in clinical trials are illustrated in **Table 1** (18).

**Table 1:** Major design methods and terminology commonly employed in adaptive clinical trials (18).

Adaptive trial types and special topics	Description in brief
Group sequential design	Prospective interim analysis with pre-specified criteria for terminating the trial
Sample size	Prospectively planned modifications to the sample size based on interim estimates (e.g. unblinded sample size adaptation/re-estimation)
Patient population (e.g. adaptive enrichment)	Adaptive modification of the patient population based on comparative interim results
Treatment arm selection (pick-the-winner/drop-the-loser; adaptive dose ranging, etc.)	Adding or terminating arms (dropping the inferior treatment group(s), modifying treatment arms and/or adding additional arms; to allocate more patients to treatment doses of interest, reducing allocation of patients to doses that appear non-informative, etc.)
Patient allocation (randomization or treatment switching, etc.)	Adaptation based either on comparative baseline characteristics or based on comparative outcome data
Endpoint selection	Adaptive modification to the choice of primary endpoints based on comparative interim results
Seamless Phase II/III	Allows combined objectives (Phase II and III) moving from Phase II (investigational stage) to Phase III (efficacy or confirmatory) without stopping the patient enrolment process
Biomarker adaptive	Allows adaptations based on an interim analysis of the treatment responses of biomarkers (can be used to select patient populations for subsequent trials, identify the natural course of a disease)
Multiple design features	Combination of two or more adaptive features (complex)
*Special considerations and topics*	Simulation in AT planning Bayesian design Time-to event setting Potential surrogate or intermediate endpoints Secondary endpoints and safety considerations Design changes/hypothesis change

In the case of rare cancers, recruiting sufficient numbers of patients that can be analyzed with conventional statistical techniques and reject the null hypothesis can be difficult, if not impossible, to achieve (21). To address this, adaptive trials in rare cancers can be formulated using Bayesian methods. While these do not ensure smaller sample sizes, they allow prior information (e.g. from previous or historical trials, scientific research) to be combined with information accrued during a trial, as well as with the usual data available on completion of the trial, to provide probabilities that the clinical effect lies in a particular range (22, 23). This approach provides probabilities of treatment effects that apply directly to the next patient who is similar to those treated in any completed or ongoing trial (21). By supplementing the restricted information from the trial itself, the Bayesian approach has particular advantages in rare diseases. Randomized controlled trials using adaptive designs and Bayesian methodology feature highly in trials from the International Rare Cancers Initiative (IRCI), a partnership that aims to stimulate and facilitate the development of international clinical trials for patients with rare cancers (24).

### Surrogate Clinical Endpoints

In many cancers, the true outcome of interest and importance is overall survival, an outcome that is also highly endorsed by European Society for Clinical Oncology (ESMO) and American Society of Clinical Oncology (ASCO) frameworks for assessing the clinical value of cancer treatment options (25, 26). However, it can take a long time and a large patient population to assess whether a new InMP improves overall survival (27), neither of which may be an option in rare cancer trials. Surrogate markers are intermediate endpoints that serve as substitutes for direct measures of how patients feel, function, or survive in clinical trials. If an alternative endpoint is sufficiently highly correlated with overall survival, it can be used as a surrogate. For example, progression-free survival, which is the duration of time between starting medication therapy and the progression of the disease (e.g. an increase in the size or extent of the tumor), is often used as a surrogate endpoint for overall survival in oncology. Other known surrogate endpoints include pathological response rate or pathological complete response rate, event-free survival, disease-free survival, and objective response rate (ORR) (28). In a study of surrogate endpoints in metastatic breast cancer, the median overall survival was 21.6 months compared with a median duration of 7.1 months for progression-free survival (29). Far less time is therefore required to measure the effect of the new medication on the surrogate outcome, creating an opportunity to significantly reduce sample size, duration and cost of clinical trials.

The FDA has strict criteria for approving the use of a surrogate as the primary endpoint. The main criteria are that the surrogate outcome has a credible clinical relationship with the true outcome, and is highly predictive of the true outcome (30). In order to demonstrate validity, a high correlation between effects on the surrogate and the true outcome of interest is required. FDA guidance states that where a potential surrogate endpoint exists that is correlated with the primary endpoint, and the primary endpoint itself is difficult or slow to ascertain, an adaptive design can be based on the potential surrogate endpoint. For example, in a trial with a primary endpoint of overall survival in which median survival time is well over 2 years and tumor response (e.g. complete or partial response) is anticipated to predict clinical benefit, adaptive features such as sample size reassessment could be based on tumor response rather than mortality (18). The final evaluation of efficacy would still be based on the primary endpoint (overall survival in this example).

## Literature Search of Adaptive Trials in Rare Cancer

To review the types of registered adaptive trials being used in clinical trial research in rare cancer, a search of the ClinicalTrials.gov database (31) was performed covering the period March 2000 to September 2020. The classification of adaptive trials in the literature is inconsistent making their retrieval difficult and potentially underestimating their use (32). Furthermore, in the clinical trial description, many rare cancers are described by their name only and not as rare. The use of the search term ‘rare cancer’ is therefore likely to significantly underestimate the true number of trials.

To capture as many ongoing and completed trials as possible, the authors supplemented search terms used in a previous review of adaptive clinical trials (10) to create the following list of descriptions commonly-used in adaptive trial designs: adaptive dose, adaptive endpoint, adaptive group sequential, adaptive randomization, adaptive sample size, adaptive seamless, adaptive switching, adaptive treatment group, biomarker adaptive, multi-arm multi-stage, pick the winner/drop the loser, basket trial, platform trial, and umbrella trial. Two authors (AK and MZ) confirmed whether the identified trials were adaptive in design and that the indication was in rare cancer.

A previous review of the medical literature has shown that trial design nomenclature is not standardized and the application of the adaptive methodology is often not reported explicitly as ‘adaptive design’ and has to be inferred from the trial description making it more difficult to capture relevant trials (33). This was also the case in the current study where none of the 72 trials identified in the ClinicalTrials.gov database using the search term ‘rare cancer’ incorporated ‘adaptive design’ anywhere in their description. An adaptive design was inferred in nine trials. A further eight rare cancer trials with an adaptive design were identified by searching all oncology trials and checking the cancer description to determine if it was rare. The list of trials identified, and the type of adaptive design used is shown in **Supplementary Table 2**. The majority of trials were Phase II or Phase III and used ORR as the primary endpoint. Enrolment sizes ranged from 21 to 770. From the limited clinical trial data available, there appeared to be no preferred type of adaptive trial format for the rare cancer trials.

By changing the search from ‘rare cancer’ to ‘oncology’, 106 trials using an adaptive design could be identified. The most frequently used adaptive trial methodologies were adaptive treatment group, adaptive dose, adaptive randomization, adaptive endpoint, and biomarker adaptive (**Supplementary Table 3**).

## Clinical Value of Adaptive Trials in Rare Cancer

### An Online Survey of Oncologists Practicing in Rare Cancer

Wider implementation of adaptive trials, particularly in rare cancers, requires a better understanding of their value for clinical decisions for patients by practicing oncologists. Some of the barriers to their use include unfamiliarity and confusion surrounding the different types of adaptive trial design and the statistical methods required, and fear of jeopardizing chances of regulatory approval. To overcome some of these barriers a set of CONSORT guidelines has been developed specifically for publishing adaptive trials (34).

**Figure 1:**
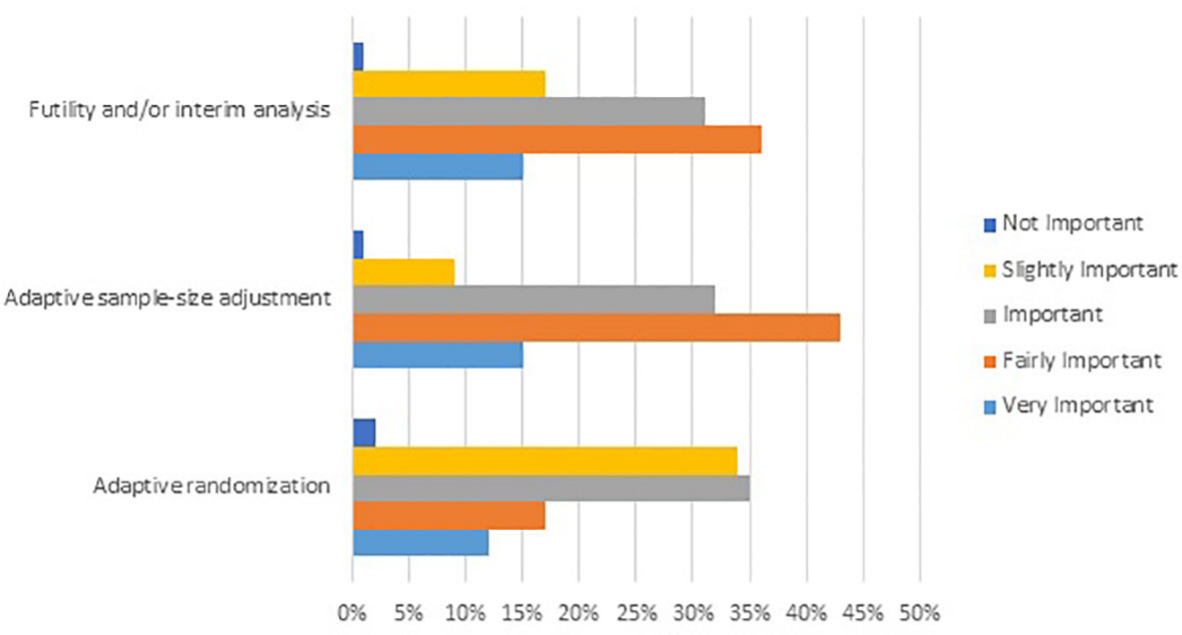
Results from an online survey showing the level of importance attributed by rare cancer oncologists to selected Phase II/III adaptive trial designs for clinical decisions in rare cancer. Survey question: Please select the level of importance for each attribute of the following adaptive trial designs when evaluating clinical evidence for an innovative medicinal product in rare cancer (N=231).

To further explore the understanding and acceptance of adaptive trials among clinicians practicing in rare cancer an on-line survey was initiated in March 2020. Three thousand oncologists were contacted to participate using a mainstream survey platform. Respondents were sent an e-mail explaining the purpose of the survey and a link to the actual survey platform. Participants were questioned on the importance of different evidence levels, types of adaptive trial design, and categories of surrogate endpoints for clinical decisions in rare cancers (**Figure 1**, **Supplementary Figures 1A**, **B**). Attributes were rated on a five-point scale from 1 not important/strongly disagree to 5 very important/strongly agree. Due to stringent regulations related to data privacy protection in the European Union, no personally identifiable data was requested or captured in the survey, and there was no tracking of individual participant completion. Only fully completed responses were included in the analysis. The results of the survey were accepted as a poster presentation at ESMO 2020 (35).

A total of 231 surveys were returned complete. Most respondents were from North America and Europe (42% and 38%, respectively) with small representations from Asia and Australia (14% and 6%, respectively). The majority of the responders were oncologists specializing/practicing in medical or clinical oncology and rare cancer (74%).

For clinical decisions in rare cancer, evidence from Phase II/III trials with an adaptive design (even with relatively small sample size) was rated highly, even more so than evidence from conventional randomized controlled trials (97% and 82% of responders, respectively). Among the different types of adaptive design, futility analyses, and/or interim analyses, adaptive sample size adjustment and adaptive randomization were perceived as more important than seamless design or adaptive patient population (**Figure 1**).

Surrogate endpoints, such as time-to-first-subsequent treatment (TFST), time-to-second progression (PFS2), or time-to-second-subsequent treatment (TSST), along with progression-free survival, were considered valuable alternatives to overall survival by the majority of oncologists (**Supplementary Figures 1A**, **B**).

## Discussion

Regulatory agencies worldwide have instituted procedures for fast-track approval of InMPs for rare disorders and serious diseases, but it is recognized that innovative clinical trial designs are required to explore the potential of new agents in rare cancers. These should include the use of adaptive designs when well-powered randomized controlled trials are not feasible due to the low incidence of the inquired cancer. In many cases, this will require Bayesian approaches. The latter require sufficient information on the disease to power the statistics, highlighting the importance of clinical databases and tissue banks to provide data on rare cancers. Novel Bayesian adaptive designs continue to be explored such as Bayesian Optimal Interval (BOIN), the keyboard, the TIme-To-Event BOIN (TITE-BOIN), the BOIN combination, and the Bayesian Optimal Phase 2 (BOP2) designs (36). These have been designed to increase study efficiency, allow more flexible trial conduct, and treat a greater number of trial patients with more effective treatments. Simpler to implement than traditional Bayesian designs, they may also facilitate the use of adaptive designs in rare cancers. The rate of uptake of adaptive designs in clinical research has, however, remained well behind that of the new methods introduced in the statistical literature.

A retrospective analysis of the FDA internal database for the period December 1987 to May 2011 identified 45 oncology products approved for 68 rare cancer indications, and which were supported by 99 trials (37). One-third of the trials were randomized and 67% were single-arm, although no information was provided on whether any of the trials used adaptive designs. In agreement with our findings from the ClinicalTrials.gov website, ORR was relied on as the primary efficacy endpoint in 69% of approvals, correlating with the prevalence of single-arm trials.

While high-quality randomized controlled clinical trials remain the gold standard when evaluating the potential of a novel intervention, adaptive trials and surrogate endpoints may offer a solution for some rare cancer trials, and our survey of practicing oncologists showed that data from adaptive designs in rare cancers are regarded just as highly. Adaptive designs offer many important advantages including an earlier selection of the most promising patient characteristics or therapeutic options. Interim analysis allows the trial to be stopped early, for example, for futility, which, in turn, can help limit patient exposure to ineffective treatments. In multi-arm trials, an efficacy signal specific for one rare cancer but not others allow the latter cohorts to be stopped without any need for a protocol amendment.

In the era of personalized medicine, adaptive designs may also prove beneficial in more common cancers with specific markers that allow them to be classed as orphan subsets. For example, 4% to 7% of patients with non–small cell lung cancer (NSCLC) have overexpression of anaplastic lymphoma kinase (ALK). Crizotinib, a first-in-class multitargeted tyrosine kinase inhibitor, was specifically developed for patients with ALK-positive tumors and was granted orphan status on this basis. Several other life-threatening tumors are characterized by ALK genetic alterations and one of the adaptive design trials identified in the ClinicalTrials.gov website was a multi-center multi-arm trial with the ALK inhibitor ceritinib.

It has been hypothesized that the physiological basis for rare tumors being rare is that they are driven by a single deviant mechanism, such as a single gene anomaly (38). For this reason, such cancers should also be more treatable compared with cancer caused by numerous distinct molecular defects. Therefore, rare cancers represent an opportunity for understanding responses in other more common cancers, some of which have rare subsets identified by the expression of specific biomarkers.

InMPs are increasingly subject to scrutiny over their cost and value, particularly in markets such as oncology where the increasing prevalence of cancer is coupled with high costs of drug development. In this arena, the use of adaptive trials can offer increased value to a range of stakeholders. For the pharmaceutical company they can reduce development resources in that trials are completed with reduced timelines and at lower cost, potentially shortening the time to reach the market. Adaptive trials can also be used for a variety of development opportunities from dose finding to expanding indications and offer the opportunity for a seamless switch to a Phase III confirmatory trial if early results are favorable. Such improved value for pharmaceutical companies may subsequently encourage greater involvement in research in rare cancers and other diseases. For physicians and researchers, participation offers access to a valuable data set in terms of outcomes, imaging and biomarkers and opportunities for continuous learning. For patients and advocates, adaptive trials and surrogate endpoints increase the likelihood that patients receive the most beneficial treatment for their cancer subtype at an earlier point in their disease. Strategies to individualize treatment, for example by the identification of biomarkers to better predict treatment response, also offer value-based treatment, which is increasingly demanded by payers when making prescribing decisions.

During the writing of this paper, two important unmet needs related to the registration and reporting of adaptive clinical trials in rare cancers were identified. First, it became apparent that there is a need to harmonize procedures and definitions when reporting adaptive trials in rare cancer, both in clinical trial registers and in the medical literature. At present, this information must be inferred from the limited descriptions provided in the trials database. Whether this is due to inadequate data input by the trial coordinator and/or because registration sites do not have adequate flexibility to describe some adaptive trial features remains to be determined. The term ‘adaptive clinical trial’ is an all-encompassing label that covers a wide range of designs that vary greatly in complexity, and this is only likely to increase as new innovative adaptive designs are developed. To avoid ambiguity and improve reporting, consensus is required on adaptive design terminology and procedures so that they can be consistently used. This has been documented for certain adaptive trial designs, e.g. adaptive platform trials (39), but more widespread consensus definitions are required in this area to speed adoption of adaptive trials and to promote best practice for their use. The fact that adaptive trials are difficult to find may lead to the perception that they are not being performed. A second unmet need relates to the registration and reporting of trials in rare cancer. Only a few of the rare cancer trials identified were reported as such on clinical trial registries, instead requiring searches by specific rare cancer type. The simple addition of ‘rare cancer’ to the registry trial description or scientific publication would greatly assist future researchers in this field. To some extent this may reflect confusion over the definition of ‘rare,’ which differs between rare cancers and rare diseases, the former being based on incidence and the latter being based on prevalence. Consensus on these issues will be of value to investigators planning future research in rare cancers and other diseases and may also prove of practical use to medical societies and manufacturers to support broader implementation of adaptive designs in the development of innovative medicinal products.

## Author Contributions

AK developed concept of the research and outline of the manuscript. AK, SS, and MZ contributed equally in the analysis and interpretation of the results, development, review, and approval of the manuscript. All authors contributed to the article and approved the submitted version.

## Disclaimer

The opinions and ideas expressed in this article are personal and do not necessarily represent those of the companies.

## Conflict of Interest

AK is an employee of Apogenix AG, SS is an employee of System Analytic, and MZ is an employee of Sandoz GmbH.

## Supplementary Material

Supplementary Table 1 Advantages and disadvantages of adaptive trial designs for innovative medicinal products in rare cancers (FDA 2019 guidance) (18).

Supplementary Table 2 Results of ClinicalTrials.gov search of rare cancer trials with an adaptive design.

Supplementary Table 3 Results of ClinicalTrials.gov search of oncology trials with an adaptive design.

## References

[1] Greenlee RT Goodman MT Lynch CF Platz CE Havener LA Howe HL. The occurrence of rare cancers in US adults, 1995-2004. Public Health Rep (2010) 125:28–43. 10.1177/003335491012500106 20402194 PMC2789814

[2] Gatta G van der Zwan JM Casali PG Siesling S Dei Tos AP Kunkler I. Rare cancers are not so rare: the rare cancer burden in Europe. Eur J Cancer (2011) 47:2493–511. 10.1016/j.ejca.2011.08.008 22033323

[3] RARECAREnet. Information network on rare cancers. In: List of rare cancers (2020). Available at: http://www.rarecarenet.eu/rarecarenet/index.php/cancerlist (Accessed October 2, 2020).

[4] Gatta G Capocaccia R Botta L Mallone S De Angelis R Ardanaz E. RARECAREnet working group. Burden and centralised treatment in Europe of rare tumours: results of RARECAREnet-a population-based study. Lancet Oncol (2017) 18(8):1022–39. 10.1016/S1470-2045(17)30445-X 28687376

[5] Gatta G Trama A Capocaccia R. RARECARENet Working Group. Epidemiology of rare cancers and inequalities in oncologic outcomes. Eur J Surg Oncol (2019) 45(1):3–11. 10.1016/j.ejso.2017.08.018 29032924

[6] DeSantis CE Kramer JL Jemal A. The burden of rare cancers in the United States. CA Cancer J Clin (2017) 67:261–72. 10.3322/caac.21400 28542893

[7] Krendyukov A Gattu S. Critical factors shaping strategy development of an innovative medicine in oncology. Pharmaceut Med (2020) 34(2):103–12. 10.1007/s40290-020-00328-x 32107738

[8] Renfro LA Ji L Piao J Onar-Thomas A Kairalla JA Alonzo TA. Trial design challenges and approaches for precision oncology in rare tumors: experiences of the Children’s Oncology Group. JCO Precis Oncol (2019) 3PO.19.00060. 10.1200/PO.19.00060 PMC7446492 32923863

[9] Hilgers RD König F Molenberghs G Senn S. Design and analysis of clinical trials for small rare disease populations. J Rare Dis Res Treat (2016) 1(3):53–60. 10.29245/2572-9411/2016/3.1054

[10] Bothwell LE Avorn J Khan NF Kesselheim AS. Adaptive design clinical trials: a review of the literature and ClinicalTrials.gov. BMJ Open (2018) 8(2):e018320. 10.1136/bmjopen-2017-018320 PMC5829673 29440155

[11] Tan SB Dear KB Bruzzi P Machin D. Strategy for randomised clinical trials in rare cancers. BMJ (2003) 327(7405):47–9. 10.1136/bmj.327.7405.47 PMC1126386 12842959

[12] Small population clinical trials: challenges in the field of rare diseases (2016). Available at: https://www.irdirc.org/wp-content/uploads/2017/12/SPCT_Report.pdf (Accessed Aug 31, 2020).

[13] European Federation of Pharmaceutical Industries and Associations. The pharmaceutical industry in figures, in: Key data 2017. Available at: https://www.efpia.eu/media/219735/efpia-pharmafigures2017_statisticbroch_v04-final.pdf (Accessed Sep 30, 2020).

[14] Wouters OJ McKee M Luyten J. Estimated research and development investment needed to bring a new medicine to market, 2009-2018. JAMA (2020) 323(9):844–53. 10.1001/jama.2020.1166 PMC7054832 32125404

[15] Prasad V Mailankody S. Research and development spending to bring a single cancer drug to market and revenues after approval. JAMA Intern Med (2017) 177(11):1569–75. 10.1001/jamainternmed.2017.3601 PMC5710275 28892524

[16] Singh H Pazdur R Xu L Liu K. FDA breakthrough therapy designation for oncology products: the CBER experience. J Clin Oncol (2018) 36(15 Suppl):e18585. 10.1200/JCO.2018.36.15_suppl.e18585

[17] European Medicines Agency. Launch of PRIME—Paving the way for promising medicines for patients (2016). (Accessed Sep 15, 2020).

[18] U.S. Department of Health and Human Services Food and Drug Administration Center for Drug Evaluation and Research (CDER) Center for Biologics Evaluation and Research (CBER). Adaptive designs for clinical trials of drugs and biologics guidance for industry (2019). Available at: https://www.fda.gov/media/78495/download (Accessed Sep 20, 2020).

[19] Gallo P Chuang-Stein C Dragalin V Gaydos B Krams M Pinheiro J. PhRMA Working Group. Adaptive designs in clinical drug development–an Executive Summary of the PhRMA Working Group. J Biopharm Stat (2006) 16(3):275–83. 10.1080/10543400600614742 16724485

[20] Chow SC Chang M. Adaptive design methods in clinical trials – a review. Orphanet J Rare Dis (2008) 3:11. 10.1186/1750-1172-3-11 18454853 PMC2422839

[21] Lilford RJ Thornton JG Braunholtz D. Clinical trials and rare diseases: a way out of a conundrum. BMJ (1995) 311(7020):1621–5. 10.1136/bmj.311.7020.1621 PMC2551510 8555809

[22] Lee JJ Chu CT. Bayesian clinical trials in action. Stat Med (2012) 31(25):2955–72. 10.1002/sim.5404 PMC3495977 22711340

[23] Ryan EG Brock K Gates S Slade D. Do we need to adjust for interim analyses in a Bayesian adaptive trial design? BMC Med Res Methodol (2020) 20:150. 10.1186/s12874-020-01042-7 32522284 PMC7288484

[24] Bogaerts J Sydes MR Keat N McConnell A Benson A Ho A. Clinical trial designs for rare diseases: studies developed and discussed by the International Rare Cancers Initiative. Eur J Cancer (2015) 51(3):271–81. 10.1016/j.ejca.2014.10.027 PMC4639696 25542058

[25] Cherny NI Dafni U Bogaerts J Latino NJ Pentheroudakis G Douillard JY. ESMO-magnitude of clinical benefit scale version 1.1. Ann Oncol (2017) 28:2340–66. 10.1093/annonc/mdx310 28945867

[26] Schnipper LE Davidson NE Wollins DS Tyne C Blayney DW Blum D. American Society of Clinical Oncology statement: a conceptual framework to assess the value of cancer treatment options. J Clin Oncol (2015) 33(23):2563–77. 10.1200/JCO.2015.61.6706 PMC5015427 26101248

[27] Hernandez-Villafuerte K Fischer A Latimer N. Challenges and methodologies in using progression free survival as a surrogate for overall survival in oncology. Int J Technol Assess Health Care (2018) 34(3):300–16. 10.1017/S0266462318000338 29987997

[28] Gyawali B Hey SP Kesselheim AS. Evaluating the evidence behind the surrogate measures included in the FDA’s table of surrogate endpoints as supporting approval of cancer drugs. EClinicalMedicine (2020) 21:100332. 10.1016/j.eclinm.2020.100332 32382717 PMC7201012

[29] Burzykowski T Buyse M Piccart-Gebhart MJ Sledge G Carmichael J Lück HJ. Evaluation of tumor response, disease control, progression-free survival, and time to progression as potential surrogate end points in metastatic breast cancer. J Clin Oncol (2008) 26(12):1987–92. 10.1200/JCO.2007.10.8407 18421050

[30] ClinicalTrials.gov. a service of the US National Institutes of Health. Available at: https://clinicaltrials.gov/ (Accessed Oct 1, 2020).

[31] Guidance for Industry. clinical trial endpoints for the approval of cancer drugs and biologics: Food and Drug Administration (2007). Available at: http://www.fda.gov/downloads/Drugs/GuidanceComplianceRegulatoryInformation/Guidances/ucm071590.pdf (Accessed Sep 24, 2020).

[32] Hatfield I Allison A Flight L Julious SA Dimairo M. Adaptive designs undertaken in clinical research: a review of registered clinical trials. Trials (2016) 17:150. 10.1186/s13063-016-1273-9 26993469 PMC4799596

[33] Mistry P Dunn JA Marshall A. A literature review of applied adaptive design methodology within the field of oncology in randomised controlled trials and a proposed extension to the CONSORT guidelines. BMC Med Res Methodol (2017) 17(1):108. 10.1186/s12874-017-0393-6 28720094 PMC5516365

[34] Dimairo M Pallmann P Wason J Todd S Jaki T Julious SA. The Adaptive designs CONSORT Extension (ACE) statement: a checklist with explanation and elaboration guideline for reporting randomised trials that use an adaptive design. BMJ (2020) 369:m115. 10.1136/bmj.m115 32554564 PMC7298567

[35] Krendyukov A Zabransky M Singhvi S. Innovative medicine in rare cancer: perceived value of adaptive trials and surrogate endpoints for clinical decisions. Ann Oncol (2020) 31(Suppl 4):546P. 10.1016/j.annonc.2020.08.660 PMC7982798 33763370

[36] Lin R Lee JJ. Novel bayesian adaptive designs and their applications in cancer clinical trials. In: Bekker A Chen DG Ferreira JT, editors. Computational and Methodological Statistics and Biostatistics. Switzerland: Springer (2020). p. 395–426. 10.1007/978-3-030-42196-0_17

[37] Gaddipati H Liu K Pariser A Pazdur R. Rare cancer trial design: lessons from FDA approvals. Clin Cancer Res (2012) 18(19):5172–8. 10.1158/1078-0432.CCR-12-1135 22718862

[38] Braiteh F Kurzrock R. Uncommon tumors and exceptional therapies: paradox or paradigm? Mol Cancer Ther (2007) 6(4):1175–9. 10.1158/1535-7163.MCT-06-0674 17431100

[39] The Adaptive Platform Trials Coalition Angus DC Alexander BM Berry S Buxton M Lewis R Paoloni M. Adaptive platform trials: definition, design, conduct and reporting considerations. Nat Rev Drug Discov (2019) 18:797–807. 10.1038/s41573-019-0034-3 31462747

