# Supplementary material for: Value of Adaptive Trials and Surrogate Endpoints for Clinical Decision-Making in Rare Cancers
Source: Front Oncol. 2021 Mar 8;11:636561. doi: 10.3389/fonc.2021.636561 (PMC7982798; doi:10.3389/fonc.2021.636561)
Supplement: Supplementary Table 1 — Advantages and disadvantages of adaptive trial designs for innovative medicinal products in rare cancers (FDA 2019 guidance) (18). [file Table_1.docx]

**Value of Adaptive Trials and Surrogate Endpoints for Clinical Decision-Making in Rare Cancers**

Andriy Krendyukov, MD,*^1^ Sanjay Singhvi, MD,^2^ Markus Zabransky, MD^3^

**Supplementary material:**

**Supplementary Table 1**: Advantages and disadvantages of adaptive trial designs for innovative medicinal products in rare cancers (FDA 2019 guidance) (18).

| **Potential advantages** | **Potential limitations** |
| --- | --- |
| Statistical efficiency (greater chance of detecting a drug effect at a given sample size) | Specific analytical methods to avoid erroneous conclusions and/or introducing bias |
| Ethical considerations (ability to stop the trial if data are not consistent with an effective drug [futility] or if there is persuasive evidence of an important effect) | Gains in efficiency in some may be offset by losses in others |
| Improved understanding of drug effects | Efficiency gains through adaptation may be limited by important scientific constraints or in certain clinical settings |
| Acceptability to stakeholders | Logistical challenges to ensuring trial conduct and integrity |
|  | Challenges in interpretability of results |

**Supplementary Table 2**: Results of ClinicalTrials.gov search of rare cancer trials with an adaptive design.

| **ClinicalTrial.gov identifier** | **Type of adaptive trial (inferred from trial description)** | **Trial phase** | **Cancer type(s)** | **Estimated enrolment** | **Primary endpoint** |
| --- | --- | --- | --- | --- | --- |
| NCT03012620 | Multi-arm multi-stage | II | Selected rare cancers | 350 | ORR |
| NCT02465528 | Multi-arm, multi-stage | II | ALK+ tumors | 22 | DCR |
| NCT02305654 | Multi-arm multi-stage, sequential randomization | III | Squamous cell carcinoma of the penis | 400 | OS |
| NCT04116047 | Multi-arm, multi-stage | III | Oropharyngeal cancer | 695 | OS |
| NCT02034110 | Biomarker enriched | II | BRAF V600E- Mutated Rare Cancers | 206 | ORR |
| NCT04482309 | Biomarker enriched | II | HER-2 expressing tumors | 280 | ORR |
| NCT04551521 | Basket trial | II | Rare malignancies | 175 | DCR |
| NCT02372006 | Basket trial | I/II | Pediatric neuroectodermal tumors, rhabdomyosarcoma | 56 | Dose-limiting toxicity, objective response |
| NCT01110876 | Adaptive randomization | I/II | Recurrent glioblastoma multiforme | 21 | Maximum tolerated dose |
| NCT04230265 | Adaptive dose | I | Hematologic and lymphatic malignancies | 45 | 1. Safety and tolerability. 2. Incidence of dose-limiting toxicities.  3. Maximum-tolerated dose |
| NCT02914405 | Adaptive dose | I | Neuroblastoma | 36 | Incidence of TEAE |
| NCT03970447 | Adaptive response platform trial | II/III | Glioblastoma | 550 | OS |
| NCT02860494 | Adaptive seamless | II/III | Facial angiofibromas | 96 | Facial Angiofibroma Severity Index |
| NCT01266031 | Pick the winner | I/II | Glioblastoma | 96 | PFS |
| NCT02942264 | Pick the winner | I/II | Recurrent anaplastic astrocytoma and glioblastoma | 53 | Maximum tolerated dose.  PFS |
| NCT02035137 | Pick the winner | II | Relapsed or persistent neuroblastoma | 105 | ORR |
| NCT04423185 NYR | Platform trial | II | Advanced rare solid tumors | 770 | ORR |
| DCR, disease control rate. ORR, objective response rate. OS, overall survival. TEAE, treatment-emergent adverse events | | | | | |

**Supplementary Table 3**: Results of ClinicalTrials.gov search of oncology trials with an adaptive design.

| **ClinicalTrial. gov identifier** | **Type of adaptive trial (inferred from trial description)** | **Trial phase** | | **Cancer type(s)** | | **Estimated enrolment** | **Primary endpoint** |
| --- | --- | --- | --- | --- | --- | --- | --- |
| NCT01930292 | Adaptive dose | I | | Advanced solid tumors | | 31 | Number of participants with dose-limiting toxicities |
| NCT01711398 | Adaptive dose | I/IIa | | Advanced solid tumors | | 15 | Recommended optimal dose administered for ≥5 days. |
| NCT01037556 | Adaptive dose | I/II | | Refractory/relapsed acute leukemia | | 50 | Optimal individualized dose |
| NCT04169711 | Adaptive dose | I | | Advanced clear cell renal cell carcinoma | | 18 | 1. Number with (AEs). 2. Recommended Phase II dose |
| NCT02432235 | Adaptive dose | I | | Relapsed or refractory Hodgkin and Non-Hodgkin lymphoma | | 133 | Dose-limiting toxicities in determination of maximum tolerated dose |
| NCT01760525 | Adaptive dose | I | | Advanced solid tumors | | 51 | Incidence of Dose Limiting Toxicities |
| NCT04042051 | Adaptive dose | I | | Unresectable locally advanced or metastatic HER2-positive breast cancer | | 24 | Incidence of Dose Limiting Toxicities |
| NCT04164069 | Adaptive dose | IIb | | Colorectal cancer | | 20 | Recommended Phase II dose |
| NCT04455620 | Adaptive dose | I/II | | Solid tumors | | 54 | Dose limiting toxicities |
| NCT01626209 | Adaptive dose | I | | Advanced solid tumors | | 32 | Dose-limiting toxicity |
| NCT01495247 | Adaptive dose | I/II | | HER2-negative, locally advanced or metastatic breast cancer | | 18 | Dose-limiting toxicities |
| NCT03900598 | Adaptive dose | I | | Non-Hodgkin's lymphoma and chronic lymphocytic leukemia | | 180 | Dose-limiting toxicity |
| NCT02627274 | Adaptive dose | I | | Solid tumor | | 150 | Dose-limiting toxicities |
| NCT04205903 | Adaptive dose | Ib/II | | Breast cancer | | 90 | Recommended Phase II dose |
| NCT04029038 | Adaptive dose | I/II | | CD22 positive leukemia or lymphoma | | 30 | Optimal dose level |
| NCT03954704 | Adaptive dose | Ia/Ib | | Advanced solid tumors | | 213 | Dose-limiting toxicities/objective response rate |
| NCT03588650 | Adaptive dose | I | | Advanced solid tumors | | 30 | Maximum tolerated dose |
| NCT03301636 | Adaptive dose | II/III | | Unresectable or Metastatic Melanoma | | 21 | Regimen-limiting toxicities |
| NCT03122522 | Adaptive dose | II | | Metastatic melanoma | | 70 | ORR |
| NCT03063762 | Adaptive dose | Ib | | Unresectable advanced and/or metastatic renal cell carcinoma | | 69 | Dose-limiting toxicities |
| NCT03056339 | Adaptive dose | I/II | | B lymphoid malignancies | | 36 | Optimal dose |
| NCT02603445 | Adaptive dose | Ib | | Follicular lymphoma and mantle cell lymphoma | | 20 | Number with AEs |
| NCT01781572 | Adaptive dose | Ib/II | | NRAS mutant melanoma | | 102 | Dose-limiting toxicities |
| NCT01021072 | Adaptive dose | I | | Advanced cancers | | 52 | Maximum tolerated dose |
| NCT00988195 | Adaptive dose | I | | Liver cancer | | 15 | Tumor response |
| NCT02182596 | Adaptive dose | I/II | | Acute Myeloid Leukemia | | 20 | Dose-limiting toxicities |
| NCT02705859 | Adaptive dose | Ib/II | | HER2-positive breast cancer | | 19 | 1. Maximum tolerated dose. 2. Clinical benefit rate |
| NCT02669264 | Adaptive dose | I | | Acute lymphoblastic leukemia | | 35 | Dose-limiting toxicities |
| NCT03215719 | Adaptive treatment de-escalation | II | | Oropharyngeal carcinoma | | 54 | PFS |
| NCT03447470 | Multi-arm, multi-stage | I/IIa | | Advanced malignancies | | 50 | Dose-limiting toxicities |
| NCT01641939 | Multi-arm, multi-stage | II/III | | HER2-positive advanced gastric cancer | | 415 | OS |
| NCT03871036 | Multi-arm, multi-stage | I/II | | Advanced urothelial cancer | | 53 | Overall response rate (ORR) |
| NCT04159155 | Multi-arm, multi stage | II/III | | Serous or p53 abnormal endometrial cancer | | 120 | Disease free survival rate |
| NCT03317158 | Multi-arm, multi stage | I/II | | BCG-relapsing urothelial carcinoma of the bladder | | 186 | Recommended Phase II dose. 6-month relapse-free survival |
| NCT03288532 | Multi-arm, multi stage | III | | Renal Cell Carcinoma | | 1750 | DFS |
| NCT03879122 | Multi-arm, multi stage | II/III | | Metastatic hormone-sensitive prostate cancer | | 135 | OS |
| NCT00268476 | Multi-arm, multi stage | II/III | | Advancing or metastatic prostate cancer | | 12,200 | OS |
| NCT01166542 | Multi-arm, multi-stage | III | | Head and neck cancers | | 167 | OS |
| NCT01905657 | Pick the winner | II/III | | Non-small cell lung cancer | | 1061 | OS |
| NCT02499367 | Pick the winner | II | | Triple-negative breast cancer | | 84 | PFS |
| NCT01682772 | Pick the winner | II | | Castration-resistant prostate cancer | | 148 | ORR |
| NCT03764553 | Pick the winner | II | | Esophagogastric Cancer | | 310 | PFS |
| NCT02607202 | Pick the winner | II | | Cancer of unknown primary | | 120 | ORR |
| NCT04522544 | Pick the winner | II | | Hepatocellular carcinoma | | 84 | ORR |
| NCT03474094 | Pick the winner | II | | Soft tissue sarcomas | | 22 | Pathological impact |
| NCT04083651 | Futility analysis | II/III | | Advanced pancreatic cancer | | 720 | OS |
| NCT01582152 | Adaptive randomization | I/II | | Recurrent glioblastoma | | 12 | PFS |
| NCT02981342 | Adaptive randomization | II | | Pancreatic ductal adenocarcinoma | | 106 | Disease control rate (DCR) |
| NCT02814669 | Adaptive randomization | Ib | | Metastatic castration-resistant prostate cancer | | 45 | Dose-limiting toxicities |
| NCT04224493 | Adaptive biomarker | III | | Relapsed/refractory follicular lymphoma | | 518 | PFS |
| NCT03335540 | Adaptive biomarker | I | | Advanced solid tumors | | 50 | Qualified tumor biopsy specimen |
| NCT04151277 | Adaptive biomarker | II | | Metastatic pancreatic cancer | | 500 | PFS |
| NCT02664935 | Adaptive biomarker | II | | Non-small cell lung cancer | | 569 | OR and PFS |
| NCT01975142 | Adaptive biomarker | II | | HER2-negative metastatic breast cancer | | 155 | Tumor response rate |
| NCT02685397 | Adaptive biomarker | II/III | | Castration-resistant prostate cancer | | 130 | PFS |
| NCT04140162 | Adaptive response | II | | Multiple myeloma | | 50 | Achievement of minimal residual disease (MRD) negativity |
| NCT03630120 | Adaptive response | II | | Thyroid cancer | | 6 | Time to tyrosine kinase inhibitor (TKI) treatment discontinuation due to progressive disease |
| NCT01347788 | Adaptive response | I | | Castration-resistant prostate cancer | | 34 | Partial response in bone scan |
| NCT04388839 | Adaptive response | II | | Rhabdomyosarcoma | | 28 | First strike event free survival |
| NCT02753127 | Adaptive treatment group | III | | Metastatic colorectal cancer | | 1253 | OS |
| NCT02564744 | Adaptive treatment group | II | | B cell lymphomas | | 101 | 1. Number with AEs.  2. ORR |
| NCT01887522 | Adaptive treatment group | II | | Low grade gliomas | | 114 | PFS |
| NCT01555710 | Adaptive group sequential | III | | Small cell lung cancer | | 548 | OS |
| NCT03227328 | Group sequential response adaptive | II | | Advanced breast cancer | | 150 | PFS |
| NCT04128696 | Adaptive seamless | II/III | | Head and Neck Squamous Cell Carcinoma | | 600 | OS |
| NCT04428333 | Adaptive seamless | II/III | | Head and Neck Squamous Cell Carcinoma | | 640 | OS |
| NCT02529774 | Adaptive seamless | II/III | | Colorectal cancer | | 432 | OS |
| NCT04539808 | Adaptive switching | II | | Resectable or borderline resectable pancreatic cancer | | 40 | Proportion with R0 section |
| NCT03903835 | Outcome-adaptive randomization, based on observed PFS within biomarker signatures | III | | Castration-resistant prostatic cancer | | 750 | PFS |
| NCT03631706 | Adaptive recruitment | III | | Non-small Cell Lung Cancer | | 584 | PFS |
| NCT03126435 | Adaptive endpoint | III | | Adenocarcinoma of the Pancreas | | 218 | OS |
| NCT04174716 | Basket trial | I/II | | Patients with HRR Mutated Solid Tumors | | 310 | ORR |
| NCT03982173 | Basket trial | II | | Metastatic Solid Tumors | | 88 | Objective tumor response rate |
| NCT02509507 | Basket trial | Ib/II | | Advanced solid tumors | | 244 | Dose-limiting toxicity |
| NCT02428270 | Basket trial | II | | Advanced Pancreatic Cancer | | 16 | Patients with complete response, partial response, or stable disease |
| NCT04534283 | Basket trial | II | | Tumors with pathogenic alterations in BRAF, RAF1, MEK1/2, ERK1/2, and NF1 | | 35 | ORR |
| NCT03834220 | Basket trial | II | | Solid tumors harboring a fusion of FGFR1, FGFR2 or FGFR3 | | 63 | ORR |
| NCT02478320 | Basket trial | II | | CDKN2A-deficient advanced solid cancers | | 12 | Response rate |
| NCT02399943 | Basket trial | II | | RAS/RAF wild type advanced colorectal cancer | | 14 | Response rate |
| NCT04276376 | Basket trial | II | | DNA repair-deficient or platinum-sensitive solid tumors | | 1000 | ORR |
| NCT02506517 | Basket trial | II | | Multiple malignancies with HER gene aberrations | | 30 | 1. Complete response.  2. Partial response |
| NCT03872427 | Basket trial | II | | Pathway aberrant tumors (MPNST, NF1, KEAP1/NRF2 & STK11/ LKB1 | | 108 | ORR |
| NCT03518606 | Basket trial | I/II | | Advanced solid tumors | | 150 | Maximum tolerated dose and Phase II recommended dose |
| NCT03428802 | Basket trial | II | | Advanced solid tumors and genomic instability | | 40 | Response rate |
| NCT02955290 | Basket trial | I/II | | Advanced non-small cell lung cancer or squamous head and neck cancer | | 181 | 1. Dose-limiting toxicity.  2. OS |
| NCT04357873 | Basket trial | II | | Advanced mucosal cancers | | 111 | ORR |
| NCT04400474 | Basket trial | II | | Neoplasms of the endocrine system | | 144 | ORR |
| NCT03386721 | Basket trial | II | | Advanced and/or metastatic solid tumors | | 322 | Complete or partial response according to RECIST |
| NCT03915678 | Basket trial | II | | Multiple solid tumors | | 247 | Antitumor activity |
| NCT04122625 | Basket trial | II | | Multiple solid tumors | | 72 | ORR |
| NCT03297606 | Basket trial | II | | Lymphoma, Non-Hodgkin multiple myeloma, advanced solid tumors with gene aberrations in common | | 720 | ORR |
| NCT04266912 | Basket trial | I/II | | Advanced solid tumors harboring aberrations in DDR genes | | 36 | Dose-limiting toxicities. Maximum tolerated dose |
| NCT02568267 | Basket trial | II | | Solid tumors harboring NTRK 1/2/3 (Trk A/B/C), ROS1, or ALK gene rearrangements | | 300 | ORR |
| NCT04395989 | Umbrella trial | II | | Triple-negative breast cancer | | 138 | PFS |
| NCT03267589 | Umbrella trial | | II | | Relapsed ovarian cancer | 75 | Disease control rate |
| NCT02951091 | Umbrella trial | | II | | Gastric cancer | 400 | PFS |
| NCT00409968 | Umbrella trial | | II | | Non-small cell lung cancer | 341 | Biomarker profile assessment |
| NCT03292250 | Umbrella trial | | II | | Head and neck neoplasms | 259 | Disease control rate |
| NCT03574402 | Umbrella trial | | II | | Non-small-cell lung carcinoma | 400 | Response rate |
| NCT04524871 | Umbrella trial | | I/II | | Advanced liver cancers | 100 | ORR |
| NCT03424005 | Umbrella trial | | I/II | | Triple negative breast cancer | 280 | ORR |
| NCT04215003 | Umbrella trial | | Ib/II | | Breast cancer | 20 | Pathologic complete response |
| NCT04229004 | Platform trial | | III | | Metastatic pancreatic cancer | 825 | OS |
| NCT03835533 | Platform trial | | I | | Castration-resistant prostate cancer | 45 | Adverse events |
| NCT03827837 | Platform trial | | II | | Advanced urinary system and gynecological tumors | 265 | ORR |
| NCT00635193 | Platform trial | | I/II | | Ovarian cancer, primary peritoneal cancer | 138 | Efficacy and safety |
