# Supplementary material for: Value of Adaptive Trials and Surrogate Endpoints for Clinical Decision-Making in Rare Cancers
Source: Front Oncol. 2021 Mar 8;11:636561. doi: 10.3389/fonc.2021.636561 (PMC7982798; doi:10.3389/fonc.2021.636561)

Value of Adaptive Trials and Surrogate Endpoints for Clinical Decision-Making in Rare Cancers

Andriy Krendyukov, MD,*[1] Sanjay Singhvi, MD,[2] Markus Zabransky, MD[3]

Supplementary Figure 1A and Figure 1B

Supplementary Figure 1A. Level of importance of evidence attributed to time-to-first-subsequent treatment (TFST), time-to-second progression (PFS2), and time-to-second-subsequent treatment (TSST) as appropriate alternatives in the absence of progression-free-survival (PFS) or overall survival (OS) data when making clinical decisions for an innovative medicinal product in rare cancers. Survey question: Please select the level of importance attributed to each surrogate endpoint when evaluating clinical evidence for an innovative medicinal product in rare cancer (N=231).

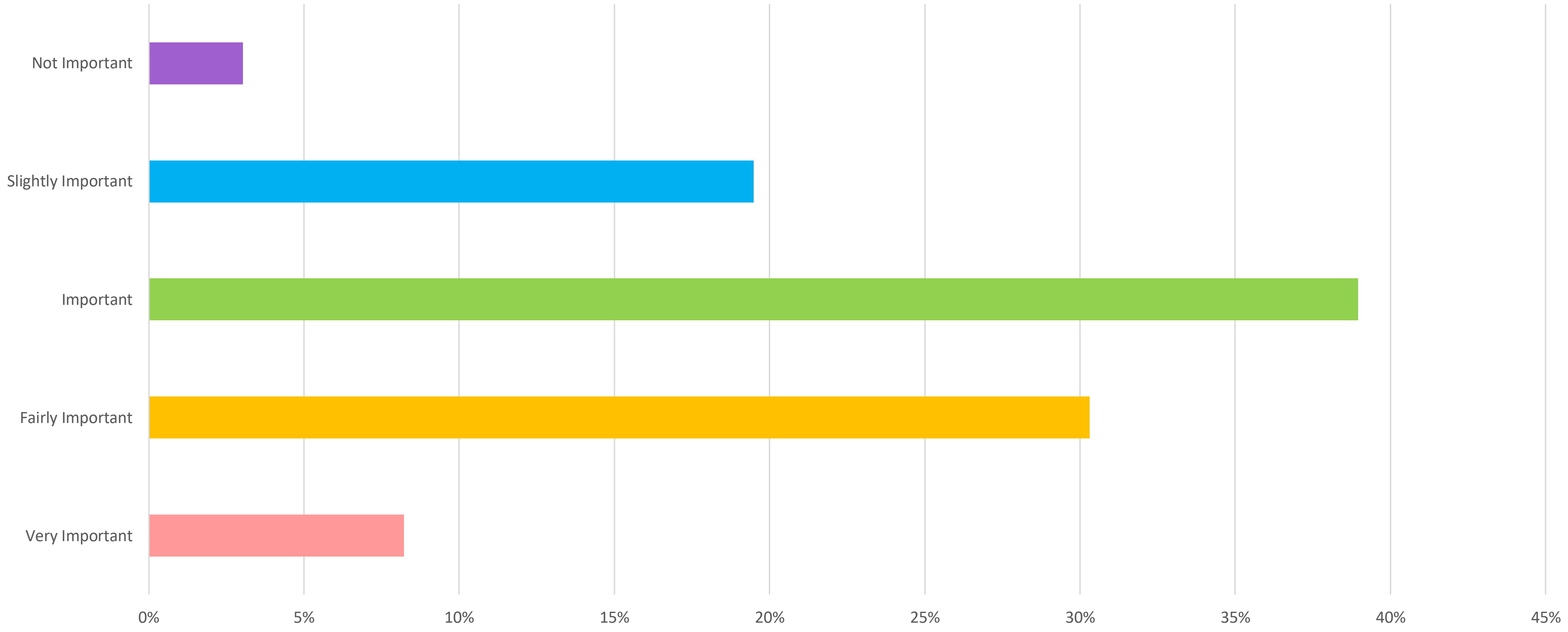

Supplementary Figure 1B. Level of importance of evidence attributed to progression-free-survival (PFS) as an appropriate alternative for clinical decision making for innovative medicinal products in rare cancers in the absence of overall survival (OS) data (N=231).

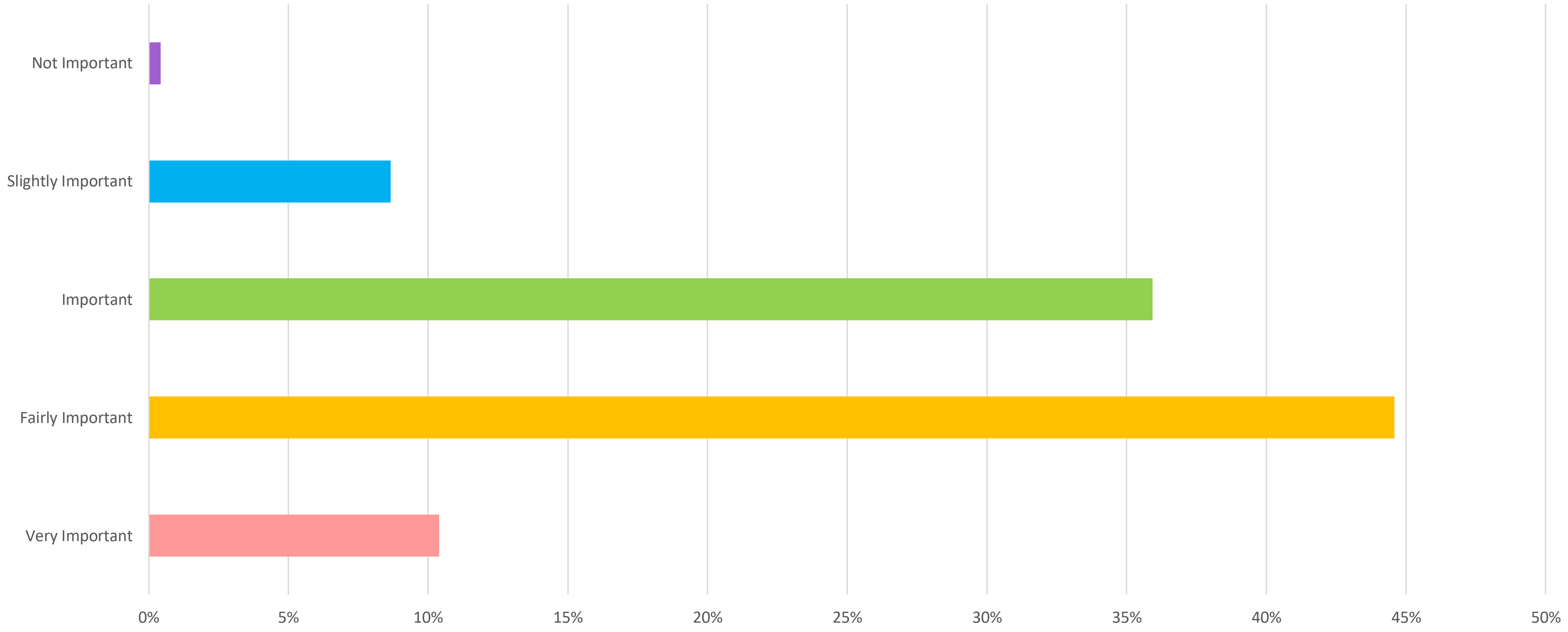

Supplement: Supplementary file 2 [file Presentation_1.pdf]
